# Giving Voice to the Environment as the Silent Partner in Aging: Examining the Moderating Roles of Gender and Family Structure in Older Adult Wellbeing

**DOI:** 10.3390/ijerph17124373

**Published:** 2020-06-18

**Authors:** Michal Isaacson, Ashwin Tripathi, Tannistha Samanta, Lisa D’Ambrosio, Joseph Coughlin

**Affiliations:** 1 Gerontology Department, University of Haifa, Haifa 3498838, Israel; 2 Indian Institute of Technology, Gandhinagar, Gujarat 382355, India; ashwin.tripathi@iitgn.ac.in (A.T.); tannistha@iitgn.ac.in (T.S.); 3 AgeLab, Massachusetts Institute of Technology, Cambridge, MA 02139, USA; dambrosi@mit.edu (L.D.); coughlin@MIT.EDU (J.C.)

**Keywords:** spatial mobility, wellbeing, older adults, family structure, gender, India

## Abstract

Gerontological scholarship has long seen the environment to be a silent partner in aging. Environmental Gerontology, an established approach in Social Gerontology, has shown how the everyday lives of older adults are deeply entangled in socio-spatial environments. Adopting an Environmental Gerontology approach, we explore social and cultural dimensions of the association between out-of-home mobility and wellbeing among older adults in a north western city of India. This was established by combining high resolution time-space data collected using GPS receivers, questionnaire data and time diaries. Following a multi-staged analytical strategy, we first examine the correlation between out-of-home mobility and wellbeing using bivariate correlation. Second, we introduce gender and family structure into regression models as moderating variables to improve the models’ explanatory power. Finally, we use our results to reinterpret the Ecological Press Model of Aging to include familial structure as a factor that moderates environmental stress. Findings emphasize the central role that social constructs play in the long-established relationship between the environment and the wellbeing of older adults.

## 1. Introduction

Environmental Gerontology, or a Gero-geographical approach, opens the doors for discussion on the convergence of aging, wellbeing, and environment. More current scholarship has introduced the broader context of society and culture into the discussion on environment and aging [1,2,3]. Although gerontology has often organized its theories around activity and wellbeing, space or spatial behavior remain neglected in its early theoretical articulations. This neglect is more intensified in the context of developing countries (notable exceptions include [4,5,6]). For instance, the last few decades in social gerontology have been dominated by the Successful Aging paradigm that defines ‘success’ within the narrow parameters of low disease probability or disease-related disability, high levels of physical and cognitive functioning, and active engagement in life. Similarly, the much earlier Activity Theory [7] puts forward a positive relationship between levels of activity and life satisfaction. Proponents of Activity Theory (Lemon, Bengston and Peterson) advocated that older adults who are active are the ones who are successful in aging. The theory draws from role theory [8] and postulates that being active leads to role enforcement, and hence, to life satisfaction. While we build on this body of work, we enter the activity–wellbeing debate through the angle of spatiality, namely spatial mobility. Specifically, we examine the role of family structure and gender in the spatial mobility of older adults by combining ecological approaches to aging with GPS-based data. 

The Ecology Model of Aging [9,10,11] helps us in understanding how Indian family structures and gender moderate the relationship between activity and quality of life or wellbeing. We examine activity through spatial mobility variables in relation to wellbeing variables, and we are in turn able to test the empirical utility of the oft-cited hypothesis—if activity theory really has a spatial dimension [6]. We start by placing the study within the broader scholarship of aging and environment in the Indian context. A brief review of the literature on environment and out-of-home activity is conducted, where activity has played a crucial role in developing gerontological literature. Our two main variables—Family structure and Gender—are then discussed, showing how these variables may influence mobility in a territorial context. Furthermore, we present our research aims, which guided the study, followed by the methods and study design section. This section explicitly discusses the study variables, our study population, sample size and wellbeing variables used. The article, ends with a discussion of the main questions raised by the analysis. In our discussion, we explore the role of territorial context in affecting the results and we draw from previous research both in developed and developing countries. 

## 2. Theoretical Framework/Literature Review

This paper examines the convergence of aging, gender, family structure and out-of-home activity. The following section touches on these different theoretical realms so that they can later be combined as the lens through which we examine our findings.

### 2.1. Aging and Environment in the Indian Context

Globally, we are experiencing growth in the number and proportion of older persons in the population. Aging is one of the most significant social transformations of the 21st century (UN, World Population Aging report 2017), with implications for family structure, housing, and transportation as well as demand in goods and services. In fact, these demographic changes are occurring at a faster rate in developing regions and, consequently, these regions are home to a growing share of world’s older population (ibid: 8). In India, the demographic bulge of the older population (although only 8.6% of the total population, Census 2011) [12] is expected to surpass the population of the United States (330 million) by 2050 in terms of absolute numbers [13]. This demographic shift will have profound implications for health, mobility and urban living—an aspect that our paper particularly focuses on. 

As aging is a global phenomenon occurring in a local context, it is beneficial to place these changes within the broader context of society and culture. In India, a majority of gerontological literature focuses on living arrangements, social security and health outcomes (see [14,15]), while some tap into descriptive studies of social capital and mobility [16,17].

Social gerontology has acknowledged the critical role of physical surroundings on older people. This acknowledgment led to the establishment of a school of thought known as Environmental Gerontology, also known as the Ecology of Aging. This is based on the foundation of Lawton and Nahemow (1973) [9], where old age is a critical phase in the life course and can be characterized by the profound influence of the physical environment. As such, Environmental Gerontology focuses on description, explanation and modification or optimization of the relation between older people and their socio-spatial surroundings [18]. Context is defined from the reference point of the developing individual throughout the lifespan, and refers to the totality of the diverse range of phenomena, events and forces that exist outside the developing individual, including its sociocultural and physical aspects. This becomes crucial in interpreting the related phenomenon of aged heterogeneity and increasing variability with age. It also helps in articulating social processes as essential and integral forces in the constitution of human development. Thus, this totality of the diverse range of phenomena, events and forces that exist outside the developing individual (ibid) has become a core feature of models of aging in biology as well as those in the social and behavioral sciences [10]. Placing knowledge gleaned from data in a broader social context enriches the environmental focal point, giving voice to the environment as a silent partner in aging. 

### 2.2. Environment and Out-of-Home Activity

Active aging is based on the idea that activity/physical movement has a positive effect on the life satisfaction of older adults, as also advocated by the Activity Theory [7]. Active aging promotes active lifestyles—regardless of age, socioeconomic status, and health—to engage in all domains (spatial, physical, environmental) of life. This formed the intellectual premise for a later gerontological narrative, popularly known as the Successful Aging discourse [19]. Rowe and Kahn (1998) advocated an active lifestyle as instrumental for health and happiness. The exclusive focus on the health benefits of physical activity in older ages has given way to more interpretive research examining “physical activity as a cultural practice and personal endeavor in older age” [20]. 

These experiential ways of understanding aging offer rich insights into the structures, organization and dynamics of physical activity in context, along with the diversity of meanings that older people experience through their involvement. Moreover, older adults’ experiences of physical activity can be diverse, shaped by a variety of socioeconomic factors and lifestyle choices that cannot be separated from the wider context and culture within which they take place [20,21]. While social aspects are important, one must not neglect the physical domains that create the context in which walkability and active transport must be examined [22]. This holds similarly with the life course perspective [23], which discusses an increased appreciation of the role of social and other environmental forces in shaping the character of human development. However, a systematic analysis of factors affecting the physical activity of older adults has remained outside the Indian gerontological tradition. While acknowledging conceptual and empirical limits to the overly optimistic “successful aging” paradigm and its inattention to questions of social inequalities, health disparities and age relations [24,25], we privilege physical movement as a crucial indicator for gendered differences in wellbeing among older persons (discussed in the following sections).

### 2.3. Aging and Family Structures 

Traditionally, older adults in India live in multigenerational families, or ‘joint families’ as they are referred to in India. Studies consistently demonstrate that the family system in India remains a critical site of aging and elder care [14,26,27,28,29]. The household living arrangements of older persons differ across regions [30,31], reflecting differences in family size and sociocultural norms surrounding the intergenerational co-residence. Scholars have recognized that social relationships, especially familial relationships, are crucial factors in influencing the health and wellbeing of individuals [28]. 

Family, as shown by social scientists, not only provides biological predictors governing health risks and outcomes, but also offers a crucial site for environment and lifestyle [32]. One’s health and aging can be strongly coupled from the family system in which it occurs. By using the life course perspective to examine familial systems, one discovers that old age is not experienced in isolation but is the product of myriad life events, choices and constraints. Moreover, an individual’s experience cannot be separated from the family in which it is embedded, therefore, to explore how family reshapes one’s mobility and aging trajectory becomes crucial. 

### 2.4. Aging and Gender Roles

Old-age is characterized by significant changes for both genders. Gender differences are expressed both biologically and socioculturally in terms of gender expectations and roles [33,34]. These differences are rooted in gender-related roles, societal functions, social status and family structures [35,36,37,38,39]. Using a gendered lens to examine aging not only allows us to recognize the importance of gender as a societal construct in later life but also reveals issues around older adults that have yet to be addressed [40,41]. 

Gender acts as a complex determinant in differing patterns of roles, responsibilities, norms, values and limitations as experienced by men and women throughout the lifespan. For instance, men, whose roles are traditionally assumed to be out of the home, in the workplace and in the community, are not expected to continue working after retirement, while women, who engage more actively in running the household, continue to bear the burden of domestic chores. This implies that older women are universally more vulnerable to social, economic, and health disadvantages than older men [26]. This leads to the well-established gender gaps in health and wellbeing that do not diminish with old age [39]. Other similar studies have focused on gender roles and physical wellbeing, gender differences and functional disability [42], and the effects of mobility on wellbeing [43]. However, gender differences in the association of spatial mobility and wellbeing have yet to be explored in a manner that replicates the complex relationship between the two. 

## 3. Research Aims

The goal of this study is to examine the complex linkages between gender, familial structure, movement and wellbeing among older adults. Specifically, we focus on the variations in physical movement among older men and women from different family settings (nuclear versus multigenerational) and how they affect overall wellbeing. 

Movement variables were collected using GPS technology, yielding data collected in natural settings. In addition, questionnaires were administered to collect data to describe subjective wellbeing. Wellbeing was measured through three variables—life satisfaction, perceived general health, and perceived physical functioning. Building on Activity Theory [7], we expected to find an association between mobility and life satisfaction. We hypothesize that the association would be stronger among men and among people living in nuclear families. Time-use data from the same participants are also included in the analysis, where possible, to provide a richer description of the culturally embedded confounders of family structures and gender. 

## 4. Methods and Study Design

The study data were collected in urban Ahmedabad (in the western state of Gujarat, India) in the months of February–March 2013, using geographical positioning system (GPS) to understand everyday life and mobility of older middle and upper middle class adults. A total of 30 participants over the age of 50, comprised of 22 males and 8 females, were recruited through a call for participation in a leading English daily and a multi-staged process, including a screening questionnaire. Participants with limitations in their activities of daily living, diagnosed psychiatric conditions, and poor English language proficiency were excluded, keeping in mind the study’s research objectives. The data collection also included time-diaries of participants’ everyday lives and in-depth qualitative interviews. Interviews were complemented by survey-based data. These surveys (questionnaires) incorporated questions that captured participants’ physical and emotional wellbeing, transportation habits and background demographics. The study received ethical clearance from the MIT Committee on the Use of Human Experimental Subjects, protocol #1212005436.

### 4.1. Sample

All 30 participants (ages between 52–82 years; mean age: 64 years) belonged to middle to upper middle class families. In some cases, middle class status was self-reported by the participants, whereas in others, we determined class status based on dimensions of home ownership, occupational profiles and the English language education of our participants. Given the fuzzy and contested empirical notion of social class (see [44,45]), we deemed these combined cultural markers more valid and reliable than solely relying on income. The sample was predominantly male, with 22 male participants and 8 female participants. One-third of our total sample of 30 participants had a family member who also agreed to participate in the study. These additional family members were either spouses or adult children. The addition of a sample of family member participants offered a unique opportunity to study people at different life stages who were living in the same environment. Family members, however, were only included in the GPS tracking portion of the study and did not complete any questionnaires. Two-thirds (*n* = 20) of the participants lived in multigenerational households. The household size ranged from 2 to 10 members. In most cases, participants were either living with their wives/husbands or their children. Most participants were married (*n* = 26, 86.7%). A majority of the total participants were either retired (*n* = 15, 50%) or unemployed (*n* = 3, 10%), but 40% of them were still employed either part-time (*n* = 9, 30%) or full-time (*n* = 3, 10%). As presented in Table 1, aside from work status, there were no significant differences between men and women, but considerable differences were found among people living in nuclear and multigenerational families when considering the background variables.

### 4.2. Wellbeing Variables (Outcome Variables)

For the assessment of wellbeing-related variables, in this study, three subjective wellbeing indicators were considered. These variables were chosen to incorporate both cognitive and affective facets of general wellbeing along with health and function-related aspects of wellbeing (Table 2). These include:

‘Life satisfaction’, reported using a single item question—how satisfied they are with their life—which was then asked to be rated on a scale from 1 to 10, with 1 being least satisfied and 10 being most satisfied. 

‘Perceived general health’, asked as a single item question under the question—Compared with others your age, how would you rate your overall health? (where a = excellent and e = poor). Answers were coded on a 5-point ranking scale, with lower values indicating better health. In order to simplify the interpretation of the results, the scores were reversed so that higher scores reflected better perceived health. 

‘Perceived physical function’, measured by the question—Do you have health problems that prevent you from doing any of the things people your age normally can do?—where the responses were either yes or no.

Before running the analysis on the data, we studied the means and standard deviations in our sub-samples with regards to wellbeing variables. Men show higher life satisfaction in our study along with more dispersion across the scale, while women’s life satisfaction is closer to the mean of the group. Meanwhile, women show high perceived general health. In terms of perceived physical functioning, all women participants report no health issues preventing their daily chores. Interestingly, there were no differences between males and females (confirmed through *t*-test) in two of our wellbeing variables. Members from nuclear families show higher life satisfaction and a smaller range when compared to the multigenerational family members. However, multigenerational families have a higher score in perceived general health. In the case of perceived physical functioning, the data distribution is quite similar. 

### 4.3. Spatial Activity Variables (Independent Variables)

The spatial measures considered in this study included variables describing volumes of out-of-home activity as well as variables describing pedestrian movement. Variables associated with vehicle travel were not included. 

The study variables included were: 

(i) Volume of spatial activity

Out-of-home activity: Average time spent outside the home and average number of activity nodes visited per day.

Walking Movement: Average time spent walking per day, average walking episodes per day and average walking tracks

Spatial mobility was analyzed through volume of movement variables (as depicted in Table 3). Average time spent out-of-home was almost 7 h each day (6.46 h) for the whole sample, but varies slightly for the members of nuclear and multigenerational families, i.e., 7.7 and 5.81 h, respectively, with an average of almost 3–4 nodes visited per day (ranging from 2–8). Men spend more time out-of-home, ranging from one hour to 17 h in a week, while for women time out-of-home ranges from three hours to 11 h. Similarly, members of nuclear families spend more time out-of-home than those in multigenerational family members. 

Maximum average time spent walking per day is higher for the male subgroup (36.69 min), while for females, it is 25.71 min. For nuclear families, the average time spent walking is more (38.50 min) than multigenerational families (30.60 min). The presence of fewer family members in nuclear family households may require more out-of-home activity and physical independence, and thus, we see more spatial mobility among the nuclear families (see Discussion). Nonetheless, we do not see stark differences between the sub-samples in spatial mobility variables. One reason could be that our inclusion criteria favored participants with no health problems that might interfere with their mobility. While the gender subgroups do not show much difference in the means of different spatial variables, when analyzed through the cultural context of family structure, they provide a richer understanding of movement among the older population. 

### 4.4. Data Analysis

We adopt a two-staged analytical strategy. First, we explored the association between spatial mobility and wellbeing through bivariate correlation. Bivariate correlations exposed the connections between our dependent (wellbeing variables) and independent (spatial mobility) variables. Following this analysis, we created four regression models using gender and familial structure as moderating variables in the links between spatial behavior and wellbeing.

One of the limitations of this study is the small sample size (30 participants). While the sample size is small, however, the spatial data that we collected for each participant are robust and included seven days of high-resolution time-space data combined with time-use diary data. Because of these limitations, we have used non-parametric analysis methods as well as partially relaxed the requirements regarding achieving statistical significance (for a more detailed discussion of this, see [46]). Instead, in order to obtain new insights from our data, we have focused primarily on effect size. 

## 5. Results

In order to illustrate the association between out-of-home mobility and quality of life, each wellbeing indicator was analyzed through bivariate correlation. In most cases, moderate to strong correlations were found between spatial mobility and our wellbeing indicators. An exception to that was the average number of nodes visited per day. Interestingly, these variables affect men and women differently, as they do for people living in nuclear and multigenerational families (see Table 4. In fact, in this study, we see how these contexts offer a more nuanced understanding of the association between movement and quality of life. 

A Spearman’s rank-order correlation was run to assess the relationship between the four spatial mobility variables and life satisfaction among older adults. Among females, there was weak correlation between life satisfaction and time spent outside the home and average walking tracks. A moderate correlation was found between average number of visited nodes per day and life satisfaction and for average time spent walking per day. For males, there was moderate correlation between life satisfaction and time spent outside the home and weak correlation for average walking tracks. A moderate correlation was found between average number of visited nodes per day and life satisfaction and for average time spent walking per day.

Among nuclear families, there was moderate correlation between life satisfaction and time spent outside the home, average walking time spent walking per day, and average walking tracks. A weak correlation was found between average number of visited nodes per day and life satisfaction. On the other hand, multigenerational families had moderate correlations between number of visited nodes per day and life satisfaction and average time spent walking per day, but weak correlations for the time spent outside the home and average walking tracks.

For the whole sample, there was moderate correlation between perceived health and time spent outside the home. Males had a weak correlation for average time spent out-of-home, average time spent walking per day, and average walking tracks, but a moderate correlation for average number of visited nodes per day. On the other hand, for females, there was a moderate correlation for all spatial mobility variables—time spent outside the home, average number of visited nodes per day, average time spent walking per day, and average walking tracks (see Table 5). 

Interestingly, there was a strong correlation for average time spent outside the home with perceived health and a moderate correlation for both average number of visited nodes per day and average time spent walking per day. However, there was a weak correlation of average walking. Similarly, in multigenerational families, there was a weak correlation between all spatial variables and perceived general health—average time spent out-of-home, average time spent walking per day and average walking tracks and average number of visited nodes per day.

In our study, perceived physical function has the strongest correlation among all other wellbeing indicators (see Table 6). For the whole sample, there were moderate correlations between perceived health and time spent outside the home and average time spent walking per day, but weak correlations for average number of visited nodes per day and average walking tracks. 

There was a strong correlation for the female subgroup, especially for the average time spent out-of-home and average number of visited nodes per day, a moderate correlation with the average time spent walking per day and perceived physical functioning, and a weak inverse correlation for average walking tracks. For the male subgroup, there were moderate correlations between perceived physical functioning and all of the spatial mobility variables—average time spent out-of-home, average number of visited nodes per day, average time spent walking per day and average walking tracks. 

For the nuclear family subgroup, there was a moderate correlation between perceived physical functioning and spatial mobility—for the average time spent out-of-home and average number of visited nodes per day. However, there was no correlation for average time spent walking per day or average walking tracks. Looking only at participants who were in multigenerational households, there were negative correlations between perceived physical health and average time spent out-of-home, average time spent walking per day and average walking tracks, but no relationship with average number of visited nodes per day. 

### 5.1. Moderated Regression Models 

To examine how gender and family structures might moderate the relationships between spatial mobility and wellbeing, we constructed moderated regression models. We plotted the data on simple slope graphs using the moderation data to better elucidate the results.

#### 5.1.1. Moderation Regression Model I

To test the hypothesis that spatial mobility is associated with wellbeing among older adults, we conducted a two-step regression. In the first step, two variables were included: time spent out-of-home (independent variable) and life satisfaction (dependent variable). These variables account for a weak association, R2 = 0.002, F (1, 21) = 0.032, *p* < 0.05. 

**Figure 1:**
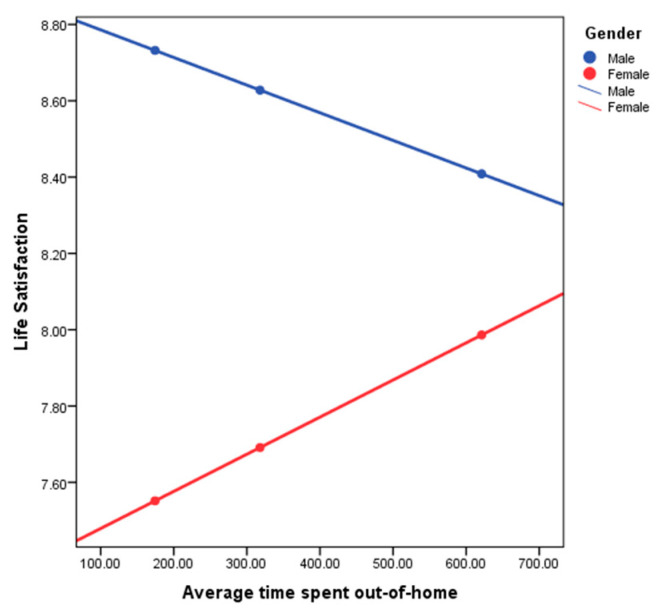
Life satisfaction with time spent out-of-home moderated by Male/Female.

In the second step, the interaction variable of gender was introduced. This improved our model fit with R2 = 0.1101, suggesting that gender accounted for 10.81% explanation of the association between life satisfaction and time spent out-of-home. Examining a simple slope plot (see Figure 1) shows how spatial behavior and life satisfaction are linked differently for men and women. 

Figure 1 depicts the direction of the relationship between life satisfaction and time spent out-of-home separately for men and women. For males (blue line), the relationship is negative (the regression line slopes downwards), whereas for females (red line), the relationship is positive (the regression line slopes upwards). The fact that the lines will cross (if extended) indicates a significant interaction effect (moderation). We can conclude that the relationship between life satisfaction and out-of-home mobility is positive for females (more time spent out-of-home associated with more life satisfaction), but negative for males (more time spent out-of-home associated with less life satisfaction). 

A similar model was analyzed by introducing the family structure as a moderator. In this case, the new model had R2 = 0.0173, suggesting that family type accounted for 1.53% of the association between life satisfaction and time spent out-of-home. 

#### 5.1.2. Moderation Regression Model II

To test the hypothesis that spatial mobility is associated with wellbeing among older adults, we conducted a two-step regression. In the first step, two variables were included: average time spent walking per day (independent variable) and life satisfaction (dependent variable). These variables account for a weak association, R2 = 0.040, F (1, 13) = 0.538, *p* < 0.05. 

**Figure 2:**
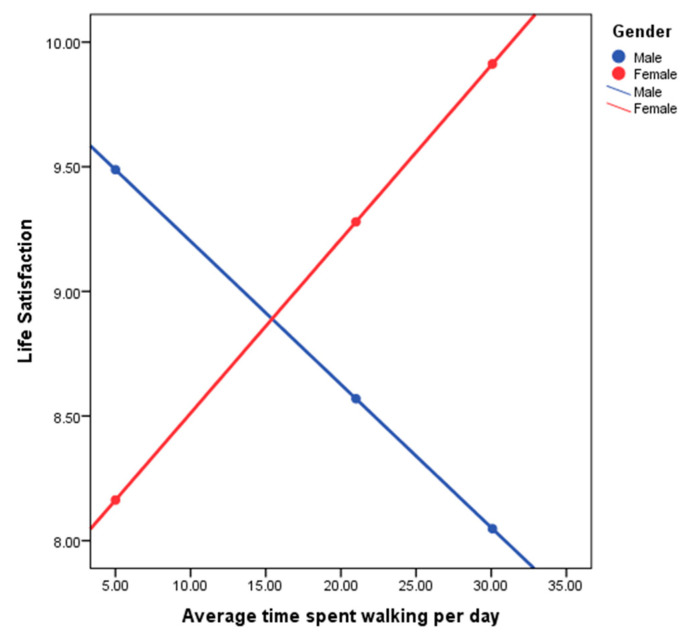
Life satisfaction with average time spent walking per day differentiated by Male/Female.

In the second step, the interaction variable of gender was introduced. This improved our model with R2 = 0.1315, leading to the understanding that gender accounted for 9.15% of the association between life satisfaction and average time spent walking per day. Examining of the simple slopes plot (see Figure 2) shows how spatial behavior and life satisfaction are linked differently for men and women. 

Examining Figure 2 demonstrates the direction of the relationship between life satisfaction and average time spent walking per day separately for men and women. For males (blue line), the relationship is negative (the regression line slopes downwards), whereas for females (red line), the relationship is positive (the regression line slopes upwards). The fact that the lines intersect indicates a significant interaction effect (moderation). We can conclude that the relationship between life satisfaction and time spent walking per day is positive for females (more average time spent walking per day associated with more life satisfaction), but negative for males (more average time spent walking per day associated with less life satisfaction). 

#### 5.1.3. Moderation Regression Model III

To test the hypothesis that spatial mobility is associated with wellbeing among older adults, we conducted a two-step regression. In the first step, two variables were included: average time spent walking per day (independent variable) and life satisfaction (dependent variable). These variables account for a weak association, R2 = 0.040, F (1, 13) = 0.538, *p* < 0.05. 

In the second step, the interaction variable of family structure was introduced. This improved our model with R2 = 0.2244, leading to the understanding that family structure accounted for 18.44% of the association between life satisfaction and average time spent walking per day. Examining of the simple slopes plot (see Figure 2) shows how spatial behavior and life satisfaction are linked differently for multigenerational and nuclear families. 

**Figure 3:**
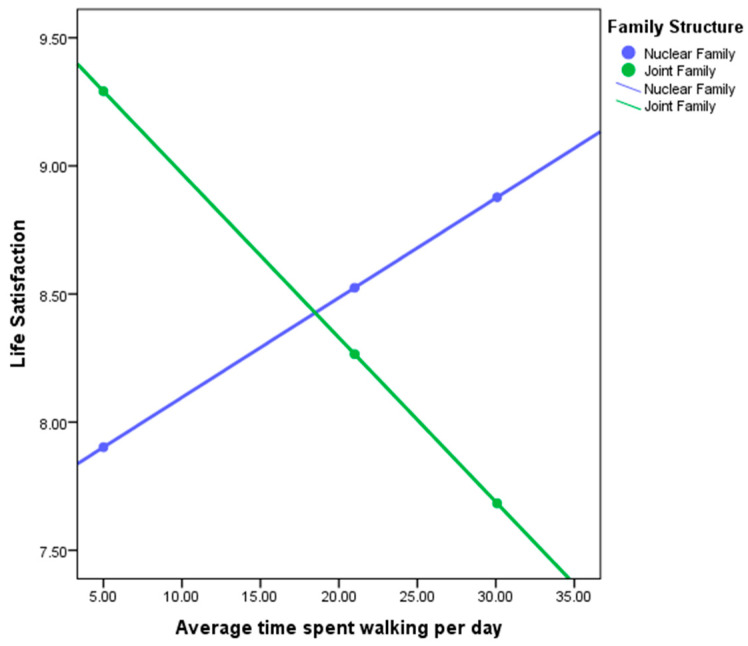
Life Satisfaction with Average time spent walking per day differentiated by multigenerational /Nuclear Family.

Examining Figure 3 demonstrates the direction of the relationship between life satisfaction and average time spent walking per day separately for men and women. For nuclear families (blue line), the relationship is positive (the regression line slopes upwards), whereas for multigenerational families (green line), the relationship is negative (the regression line slopes downwards). The fact that the lines intersect indicates a significant interaction effect (moderation). We can conclude that the relationship between life satisfaction and time spent walking per day is positive for nuclear families (more average time spent walking per day associated with more life satisfaction), but negative for multigenerational families (more average time spent walking per day associated with less life satisfaction). 

#### 5.1.4. Moderation Regression Model IV

To test the hypothesis that spatial mobility is associated with wellbeing among older adults, we conducted a two-step regression. In the first step, two variables were included: number of nodes visited per day (independent variable) and perceived general health (dependent variable). These variables account for a weak association, R2 = 0.085, F (1, 21) = 1.942, *p* < 0.05. 

**Figure 4:**
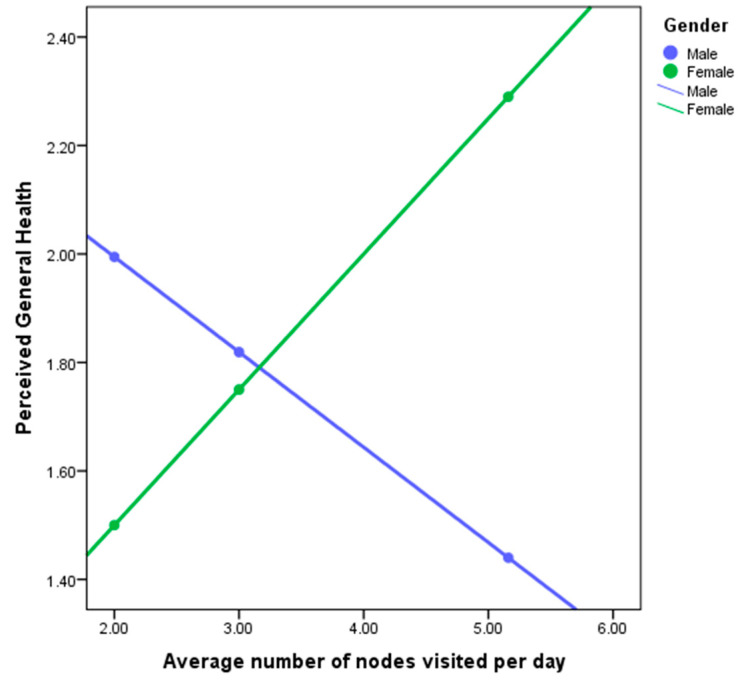
Perceived health with Average number of visited nodes per day (count)—male-females.

In the second step, the interaction variable of gender was introduced. This improved our model with R2 = 0.1267, leading to the understanding that gender accounted for 4.17% of the association between perceived general health and average number of nodes visited per day. Examining of the simple slopes plot (see Figure 4) shows how spatial behavior and perceived health are linked differently for men and women. 

Examining Figure 4 demonstrates the direction of the relationship between perceived general health and average number of nodes visited per day for men and women. For males (blue line), the relationship is negative (the regression line slopes downwards), whereas for females (green line), the relationship is positive (the regression line slopes upwards). The fact that the lines intersect indicates a significant interaction effect (moderation). We can conclude that the relationship between perceived general health and average number of nodes visited is positive for females (a greater number of nodes visited per day is associated with more perceived general health), but negative for males (a greater number of nodes visited per day associated with less perceived general health). 

A similar model was analyzed by introducing the family structure as a moderator. In this case, the new model had R2 = 0.1337, leading to the understanding that family type accounted more for 4.87% of the association between perceived general health and average number of nodes visited. With the introduction of family structure as a moderator, we see an improvement in our model.

#### 5.1.5. Moderation Regression Model V

To test the hypothesis that spatial mobility is associated with wellbeing among older adults, we conducted a two-step regression. In the first step, two variables were included: average time spent out-of-home (independent variable) and perceived general health (dependent variable). These variables have a weak association, R2 = 0.023, F (1, 21) = 0.484, *p* < 0.05. 

In the second step, the interaction variable of family structure was introduced. This improved our model with R2 = 0.1314, leading to the understanding that family structure accounted for 10.84% of the association between perceived general health and average time spent out-of-home. Examining of the simple slopes plot (see Figure 4) shows how spatial behavior and perceived health are linked differently for men and women. 

**Figure 5:**
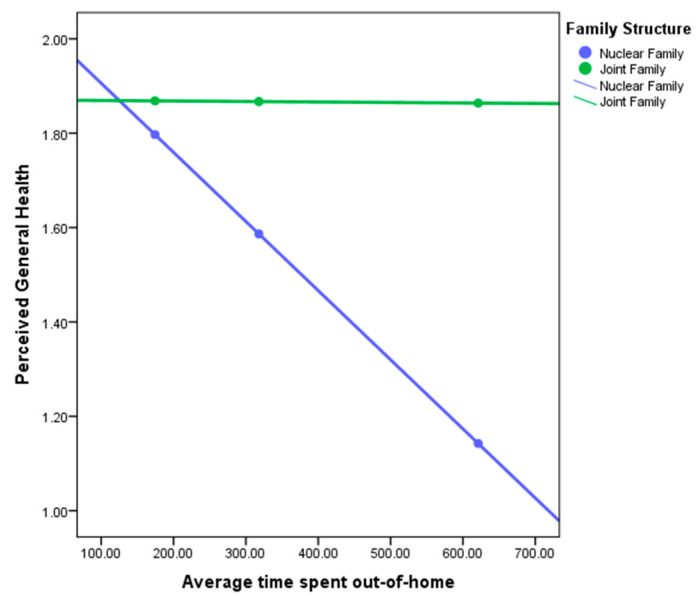
Perceived General Health with Average time spent out-of-home—Nuclear and multigenerational Family.

Examining Figure 5 demonstrates the direction of the relationship between perceived general health and average time spent out-of-home per day for nuclear and multigenerational families. For nuclear families (blue line), the relationship is negative (the regression line slopes downwards), whereas for multigenerational families (green line), the relationship goes parallel to the x-axis. We can conclude that the relationship between perceived general health and average number of nodes visited is positive for multigenerational families (more time spent out-of-home is associated with more perceived general health), but negative for nuclear families (more time spent out-of-home associated with less perceived general health). 

Similarly, we also checked how gender as a moderator affects the regression model. In this case, the new model improved from R2 = 0.023 to R2 = 0.0324. This leads us to understand that gender accounts for only 0.94% of the association between perceived general health and average time spent out-of-home. 

## 6. Time-Diary Data

We further explored the data collected using Time-Use Diaries to further illuminate our findings. As discussed before (Table 1), we see a moderate association between spatial mobility and wellbeing variables (particularly life satisfaction). The data present us with interesting empirical findings against both the Activity Theory and Role Theory. The plausible explanation was that the activities they performed were not of role supporting nature. This was clearly entered in time-use diaries, as almost 46% out-of-home activities were related to household chores, work or commercial activities. Only 14% were social activities like meeting friends and family or dining outside and only 12% recreation which included sports, leisure, and exercise. Surprisingly, only 0.04% of activities were focused on personal care. These data provided us with concrete explanation regarding the moderate relationship between spatial mobility and wellbeing. 

Moreover, this allows us to further explore activities that may have a component of role-support in our study. While these activities allowed people to remain active, they were not found to increase wellbeing. The time use data had further differences when examined on the basis of gender. Males’ out-of-home activities were comprised primarily of work, commercial purposes, household bills and religion, with small proportions devoted to exercise and family outings. On the other hand, females’ out-of-home activities included social gatherings and meeting friends and relatives, religious and education. This division of types of activity along gender lines could be assumed to have role enforcement aspects as stipulated in previous scholarly work [46,47].

In addition, in multigenerational families, we see a similar pattern of work and commercial activities involving out-of-home mobility. Many visits out-of-home involved going for a walk or exercising, and occasionally, social activities of meeting friends and relatives over meals, while in nuclear families, we see a larger mix of activities ranging from work to personal care and culture and religious activities. 

## 7. Discussion

Our analysis shows that the association between spatial mobility and wellbeing is weak when examined using bivariate analysis and simple linear regression. That is, we find limited empirical support for Activity Theory. This finding is not surprising in light of the many attempts to ground the theory using empirical results that have not fully succeeded [48]. Introducing sociocultural moderators—here, gender and family structures—adds to the degree of explanation. In what follows, we discuss the moderating effect that each has on wellbeing, and then, attempt to combine all of our findings to unpack the complex links between gender, family structure and wellbeing using spatial data.

### 7.1. Gender 

We explored differences in the association between spatial mobility and wellbeing by gender. The association between time spent out-of-home and life satisfaction increases significantly when moderated by gender. The average number of activity nodes visited by older adults is moderated in the same manner. 

Introducing gender as a moderating factor in the simple regression allows us to describe how gender influences the relationship between life satisfaction and spatial mobility more accurately. Our analysis indicates that men show a weak association while women show a stronger relationship between our study variables. This finding is counterintuitive considering our understanding of role enforcement and the ways in which men are perceived to achieve more role support out-of-home compared with the domestic aspects of female roles that may be realized in home more than out. 

Men displayed more robust out-of-home activity than women, but their activity was not associated with greater wellbeing. On further probing, through our time-use data, we discovered that the time spent out-of-home for men was primarily for work and commercial activities, while women’s out-of-home activities were more discretionary, activities over which they had more control in deciding if and how they wished to partake. Adopting this point of view suggests that choice and agency are key factors in the relationship between out-of-home mobility and wellbeing among older adults. 

Another way to understand these findings may be through the shifting of gender roles in later life or perhaps across different cohorts. Women who have significant and perhaps binding domestic responsibilities throughout the life course may be excused from these obligations as they are assumed by younger generations or as they become shared in retirement. Women freed from these obligations may have more liberty to pursue out-of-home activities that, in turn, have a positive effect on wellbeing. As noted by other scholars, age introduces a marginal erosion of expected gender roles in women’s post-reproductive lives. Older women, especially those living with daughters-in-law, attain a sense of freedom in conducting their everyday routine free of the demands of routine domestic responsibilities [49,50].

In terms of perceived general health, adding gender as a moderator variable to the regression model did not add a substantial amount of explaining power when compared to the basic linear regression model. Through descriptive analysis of spatial mobility variables, we know that women rated their overall perceived health higher than men with a low dispersion across the scale. 

### 7.2. Family

In our study, we found that family structure moderates the association between the average time spent walking per day and life satisfaction. In nuclear families, we see older adults have higher life satisfaction. We also see more time spent out-of-home and more time spent walking among the people living in nuclear families. For people living in nuclear families a moderate association exists between wellbeing and out-of-home mobility, but for those living in multigenerational families, wellbeing is inversely connected with out-of-home activity. 

**Figure 6:**
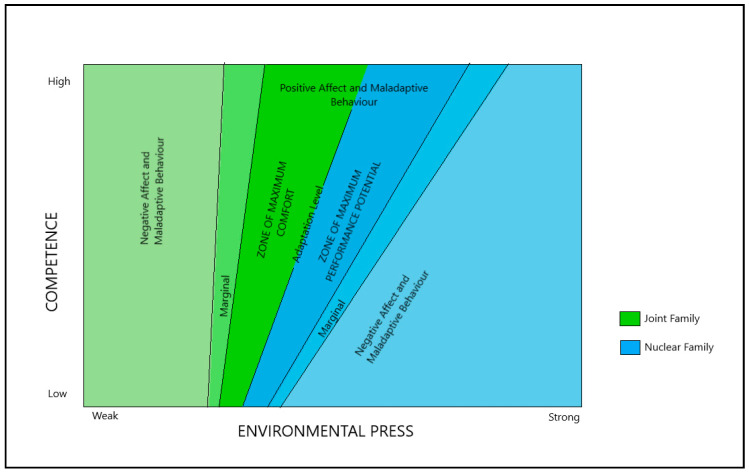
Adaption of the Ecology Model of Aging (Lawton and Nahemow 1973).

To interpret this finding, we turn to the ecological press model of aging [9,10,11]. The model stipulates that environment in later life is a component in creating positive affect. For each individual, the combination of competencies, such as physical and cognitive capabilities, and environment creates zones of maximum comfort and maximum performance and negative comfort and performance. We have extended this model to include familial structure, hypothesizing that the environmental press (stress) is higher for people living in nuclear families than it is for those living in multigenerational families (Figure 6).

We can interpret the stronger association between wellbeing and out-of-home mobility among those living in nuclear families by placing them in the zone of maximum performance, with suitable environmental press for their capabilities and for their ability to meet those demands. In multigenerational families, however, the presence of other members in the family provides a zone of maximum comfort, perhaps too much comfort, creating stagnation and possibly loss of capabilities. This is further supported by the higher mobility levels in our data among those living in nuclear families. According to the ecological press model, higher environmental press challenges, and at the same time, stimulates higher performance (mobility and life satisfaction, in this case). This may be the case in our sample of relatively young older adults. In a sample of older adults, we might find an opposite effect, as would be expected from the literature on familial structure and aging about the benefits of multigenerational families. 

Similarly, perceived general health is strongly influenced by family structures when seen in association with the average time spent outside the home. For people living in multigenerational families, there is no association between perceived health and time spent out-of-home, while people living in nuclear families displayed a negative correlation between the two variables. Two possible explanations for this may be in the robust experience of living in multigenerational family households, creating opportunities for activity and stimulation within the home. In nuclear families, out-of-home activity is a necessity for day-to-day living and not necessarily a choice or a source of enjoyment or fulfilment. 

## 8. Conclusions

This study allowed us to explore cultural factors—here, gender expectations and family structures—which possibly could affect the association between spatial mobility and wellbeing among older adults. On first examination of the literature, one might think that such a relationship would be simple. More out-of-home activity, an important aspect of being active, should be directly connected and positively corelated with wellbeing. Our current data showed us that the relationship is more complex and is influenced by social constructs. We were able to illuminate a mechanism that underlies how life satisfaction and out-of-home mobility are associated. These variables are intertwined, moderated by gender and familial structure specifically, and more generally, by social and cultural norms. In the process, we have seen that the association between spatial activity and wellbeing does not follow a simple linear trajectory but is rather embedded in one’s social and cultural environment. An important aspect of environmental exposures and health behavior is daily mobility, which has been mainly investigated in high-income countries (Sanchez et al. 2017). In this study, we focus on mobility in the global south, in the Indian subcontinent, contextualizing mobility among older adults living in Ahmedabad.

The importance of engagement and active lifestyles have been explored in depth by many scholars, but these results suggest that more cultural-specific perspectives need to be considered as well. By examining data collected in the Indian context, we are able to extend Eurocentric theories that explain the association between activity and life satisfaction in later life. This study initiates the contribution of exploring spatial dimensions into the broader discourse of activity theory and successful aging in the social gerontological literature. Moreover, this provides a way forward to explore established theories that are used to create beneficial and health promoting living environments for older adults. The contribution of this paper should be measured, including the combination of methods employed both in the different types of data that were collected for the study as well as the combined analysis methods.

### Limitations and Further Research

Further research is needed with more focus on cultural norms and variables to understand the exact mechanisms through which spatial mobility and wellbeing are related. Such work will further enhance our understanding of older adults’ lives and their wellbeing as well as broaden our findings to support better urban policies and planning as the population ages in the Global South. This study focused on a certain and specific setting, among middle class and upper middle class people living in urban Gujarat, and cannot be generalized for the whole population of the area and certainly not to all older adults in India. Data from other cultural contexts would enrich these findings, either validating them or allowing for further articulation of the cultural aspects that influence these associations.

A main limitation of this study can be found in the small sample size. Future research should include larger, more robust samples to allow for broader generalization of the results and stronger statistical significance. The results of this study should be read as insights into key themes of inquiry in an aging world and as reinforcing the importance of attending to cultural variables in understanding and explaining aging across the globe.

## Figures and Tables

**Table 1:** Sample description.

Variables	Full Sample (*n* = 30)	Gender		Test	Family Structures		Test
		Male (a) (*n* = 22)	Female (b) (*n* = 8)		Nuclear Family	Multi-Generational Family	
Age (in years) (M, SD, Range)	63.97, 8.185, 52–82	64.36, 7.85, 53–82	62.88, 9.50, 52–78	MW-Ns	65, 6.34, 53–75	63.45, 9.07, 52–82	MW-Ns
Married (*n*, %)	26, 86.66%	19, 63.3%	7, 23.3%	CS-Ns	8, 26.6%	18, 60%	CS *
Family Structure (*n*, %)	Joint Family (JF)—20, 66.7% Nuclear Family (NF)—10, 33.3%	JF–15 NF-7	JF–5 NF-3	CS-Ns	10, 33.3%	20, 66.7%	CS *
Work (*n*, %)	Unemployed—18 (60%) Employed—12 (40%)	Unemployed—10 (45.5%) Employed 12 (54.5%)	Unemployed—8 (100%)	CS-Ns	Unemployed—7 (70%) Employed—3 (30%)	Unemployed—11 (55%) Employed 9 (45%)	CS **

Note Tests: MW—Mann–Whitney U test, CS—Chi Square. Difference: “Ns” not significant; * *p* < 0.05; ** *p* < 0.01.

**Table 2:** Wellbeing Indicators including differentiation according to Gender and Family Structure.

Variable (M, SD)	Full Sample	Male	Female	Mann-Whitney	Family Structure		Mann-Whitney
					Nuclear Family	Multi-Generational Family	
Life Satisfaction	8.10, 1.82	8.23, 1.97	7.75, 1.38	Ns	8.40, 0.96	7.95, 2.13	Ns
Perceived General Health	4.20, 0.96	4.18, 1.09	4.25, 0.46	Ns	1.50, 0.70	1.95, 1.05	Ns
Perceived Physical functioning	b (no) – 25 a (yes) - 5	b – 17 (77.3%) a – 5 (22.7%)	b – 8 (100%)	Ns	b – 8 (80%) a – 2 (20%)	b – 17 (85%) a – 3 (15%)	Ns

**Table 3:** Distribution of spatial variables (Volume of Activity).

	Variables (M, SD, Range)	Full Sample (*n* = 30)	Males	Females	*t*-Test	Family Structure		*t*-Test
						Nuclear Family	Multi-Generational Family	
Volume of activity	Average time spent out-of-home (hour) per day (*n* = 23)	388.17 min, 245.102, 70–1065 min	408.50, 267.38, 70–1065	341.71, 194.73, 176–706	Ns	462.38, 175.56	348.60, 272.33	Ns
	Average number of visited nodes per day (count) (*n* = 23)	3.48, 1.563, 2–8	3.75, 1.77, 2 – 8	2.86, 0.69, 2–4	Ns	4.25, 1.98	3.07, 1.16	Ns
	Average time spent walking per day (Minute) (*n* = 15)	18.53, 11.090, 4–37	36.69, 20.86, 4–67	25.71, 12.72, 5–48	Ns	38.50, 24.11	30.60, 16.23	Ns
	Average walking tracks (count) (*n* = 23)	0.91, 1.041, 0–3	3.13, 1.78, 0–7	1.71, 0.95, 0–3	Ns	3.13, 2.53	2.47, 1.06	Ns

Note Difference: “Ns” not significant.

**Table 4:** Bivariate Correlations (Spearman correlations) between reported life satisfaction and spatial activity measures.

		Full Sample	Males (*n* = 22)	Females (*n* = 8)	Family Structure	
					Nuclear Family	Multi-Generational Family
Volume of activity	Average time spent out-of-home	−0.055	−0.191	0.099	−0.247	−0.048
	Average number of visited nodes per day (count)	−0.169	−0.208	−0.407	0.127	−0.336
	Average time spent walking per day	−0.178	−0.214	−0.527	0.286	−0.407
	Average walking tracks (count)	0.078	0.047	0.033	0.288	0.099

**Table 5:** Bivariate Correlations (Spearman correlations) between Perceived Health (overall health) and Spatial Activity.

		Full Sample	Males (*n* = 22)	Females (*n* = 8)	Family Structure	
					Nuclear Family	Multi-Generational Family
Volume of activity	Average time spent out-of-home	0.208	0.099	0.31	0.602	−0.028
	Average number of visited nodes per day (count)	0.182	0.30	−0.35	0.289	0.019
	Average time spent walking per day	−0.002	−0.09	0.27	−0.289	0.123
	Average walking tracks (count)	0.051	0.039	−0.258	0.185	−0.035

**Table 6:** Bivariate Correlation (Spearman correlation) between Perceived Physical Functioning (Health problems) and Spatial Activity.

	Perceived Physical Functioning	Full Sample	Males (*n* = 22)	Females (*n* = 8)	Family Structure	
					(0) NF	(1) MF
Volume of activity	Average time spent out-of-home	0.350	0.252	0.612	0.247	−0.045
	Average number of visited nodes per day (count)	0.086	−0.115	0.569	0.254	0
	Average time spent walking per day	0.357	0.364	0.412	0	−0.227
	Average walking tracks (count)	0.168	0.229	−0.113	0	−0.073

## References

[1] Lai M.-M., Lein S.-Y., Lau S.-H., Lai M.-L. (2016). Modeling Age-Friendly Environment, Active Aging, and Social Connectedness in an Emerging Asian Economy. J. Aging Res..

[2] Mejía S.T., Ryan L.H., Gonzalez R., Smith J. (2017). Successful Aging as the Intersection of Individual Resources, Age, Environment, and Experiences of Well-being in Daily Activities. J. Gerontol. Ser. Bpsychol. Sci. Soc. Sci..

[3] Zheng Z., Yang L. (2019). Neighborhood Environment, Lifestyle, and Health of Older Adults: Comparison of Age Groups Based on Ecological Model of Aging. Sustainability.

[4] Meijering L., Weitkamp G. (2016). Numbers and narratives: Developing a mixed-methods approach to understand mobility in later life. Soc. Sci. Med..

[5] Meijering L. (2019). Towards meaningful mobility: A research agenda for movement within and between places in later life. Ageing Soc..

[6] Isaacson M., Wahl H.-W., Shoval N., Oswald F., Auslander G. (2016). The Relationship Between Spatial Activity and Wellbeing-Related Data Among Healthy Older Adults: An Exploratory Geographic and Psychological Analysis. Cross-Cultural and Cross-Disciplinary Perspectives in Social Gerontology.

[7] Lemon B.W., Bengtson V.L., Peterson J.A. (1972). An Exploration of the Activity Theory of Aging: Activity Types and Life Satisfaction Among In-movers to a Retirement Community. J. Gerontol..

[8] Stryker S., McCall G.J., Simmons J.L. (1979). Identities and Interactions: An Examination of Human Associations in Everyday Life. Contemp. Sociol..

[9] Lawton M.P., Nahemow L. (1973). Ecology and the aging process. The Psychology of Adult Development and Aging.

[10] Wahl H.-W., Oswald F. (2012). Environmental Perspectives on Ageing. The SAGE Handbook of Social Gerontology.

[11] Wahl H.-W., Iwarsson S., Oswald F. (2012). Aging Well and the Environment: Toward an Integrative Model and Research Agenda for the Future. Gerontologist.

[12] Shetty P. (2012). Grey matter: Ageing in developing countries. Lancet.

[13] Husain Z., Ghosh D. (2016). “Analysis of Perceived Health Status Among Elderly in India: Gender and Positional Objectivity”. Cross-Cultural and Cross-Disciplinary Perspectives in Social Gerontology.

[14] Samanta T., Chen F., Vanneman R. (2014). Living Arrangements and Health of Older Adults in India. J. Gerontol. Ser. B: Psychol. Sci. Soc. Sci..

[15] Bloom D.E., Mahal A., Rosenberg L., Sevilla J. (2010). Economic security arrangements in the context of population ageing in India. Int. Soc. Secur. Rev..

[16] Samanta T., Gangopadhyay J. (2016). Social Capital, Interrupted: Sociological Reflections from Old Age Homes in Ahmedabad, India. Cross-Cultural and Cross-Disciplinary Perspectives in Social Gerontology.

[17] Isaacson M., D’Ambrosio L., Samanta T., Coughlin J. (2015). Life-Stage and Mobility: An Exploratory GPS Study of Mobility in Multigenerational Families, Ahmedabad, India. J. Aging Soc. Policy.

[18] Wahl H.-W., Weisman G.D. (2003). Environmental Gerontology at the Beginning of the New Millennium: Reflections on Its Historical, Empirical, and Theoretical Development. Gerontologist.

[19] Rowe R.L.K.J.W. (1998). Successful Aging. Gerontology.

[20] Phoenix C., Tulle E. (2017). Physical activity and ageing. Routledge Handbook of Physical Activity Policy and Practice.

[21] Gullette M. (2004). Aged by Culture.

[22] Rebecchi A., Buffoli M., Dettori M., Appolloni L., Azara A., Castiglia P., D’Alessandro D., Capolongo S. (2019). Walkable Environments and Healthy Urban Moves: Urban Context Features Assessment Framework Experienced in Milan. Int. J. Environ. Res. Public Health.

[23] Bengtson V.L., Elder G.H., Putney N.M. (2012). The life course perspective on ageing. Adult Lives.

[24] Katz S., Calasanti T. (2015). Critical perspectives on successful aging: Does it ‘appeal more than it illuminates’?. Gerontology.

[25] Lamb S. (2017). Successful Aging as a Contemporary Obsession.

[26] Knodel J., Ofstedal M.B. (2003). Gender and Aging in the Developing World: Where Are the Men?. Popul. Dev. Rev..

[27] Lamb S. (2014). Permanent personhood or meaningful decline? Toward a critical anthropology of successful aging. J. Aging Stud..

[28] Samanta T. (2019). The joint family and its discontents: Interrogating ambivalence in intergenerational relationships. Asian Popul. Stud..

[29] Visaria A., Dommaraju P. (2019). Productive aging in India. Soc. Sci. Med..

[30] Bongaarts J., Zimmer Z. (2002). Living Arrangements of Older Adults in the Developing World: An Analysis of Demographic and Health Survey Household Surveys. J. Gerontol. Ser. B Psychol. Sci. Soc. Sci..

[31] Premchand D. (2019). Living Alone in India: Gender and Policies. Glob. Soc. Secur. Rev..

[32] Umberson D., Thomeer M.B. (2020). Family Matters: Research on Family Ties and Health, 2010 to 2020. J. Marriage Fam..

[33] Cruikshank M. (2013). Learning to be Old: Gender, Culture, and Aging.

[34] Willerth M., Ahmed T., Phillips S.P., Pérez-Zepeda M.U., Zunzunegui M.V., Auais M. (2020). The relationship between gender roles and self-rated health: A perspective from an international study. Arch. Gerontol. Geriatr..

[35] Lamb S. (1997). The Making and Unmaking of Persons: Notes on Aging and Gender in North India. Ethos.

[36] Dhar H. (2001). Gender, aging, health and society. J. Assoc. Physicians India.

[37] Austen S. (2016). Gender Issues in an Ageing Society. Aust. Econ. Rev..

[38] Calasanti T., King N. (2018). The dynamic nature of gender and aging bodies. J. Aging Stud..

[39] Carmel S. (2019). Health and Well-Being in Late Life: Gender Differences Worldwide. Front. Med..

[40] Mahoney R., Calasanti T.M., Slevin K.F. (2003). Gender, Social Inequalities, and Aging. Contemp. Sociol..

[41] Venn S., Davidson K., Arber S. (2011). Gender and Aging. Handbook of Sociology of Aging.

[42] Auais M., Ahmed T., Alvarado B., Phillips S.P., Rosendaal N., Curcio C.-L., Fernandes J., Guralnik J., Zunzunegui M.V. (2019). Gender differences in four-year incidence of self-reported and performance-based functional disability: The International Mobility in Aging Study. Arch. Gerontol. Geriatr..

[43] Cuignet T., Perchoux C., Caruso G., Klein O., Klein S., Chaix B., Kestens Y. (2019). Mobility among older adults: Deconstructing the effects of motility and movement on wellbeing. Urban Stud..

[44] Fernandes L. (2006). India’s New Middle Class.

[45] Mazzarella W. (2011). Middle Class.

[46] Ahmed T., Vafaei A., Auais M., Guralnik J., Zunzunegui M.V. (2016). Gender Roles and Physical Function in Older Adults: Cross-Sectional Analysis of the International Mobility in Aging Study (IMIAS). PLoS ONE.

[47] Child S.T., Lawton L.E. (2020). Personal networks and associations with psychological distress among young and older adults. Soc. Sci. Med. (1982).

[48] Longino C.F., Kart C.S. (1982). Explicating Activity Theory: A Formal Replication. J. Gerontol..

[49] Allendorf K. (2015). Like Her Own: Ideals and Experiences of the Mother-In-Law/Daughter-In-Law Relationship. J. Fam. Issues.

[50] Samanta T., Varghese S.S. (2018). Love in the Time of Aging: Sociological Reflections on Marriage, Gender and Intimacy in India. Ageing Int..

